# You’re Beautiful When You Smile: Event-Related Brain Potential (ERP) Evidence of Early Opposite-Gender Bias in Happy Faces

**DOI:** 10.3390/brainsci14080739

**Published:** 2024-07-24

**Authors:** Jonas Schmuck, Emely Voltz, Henning Gibbons

**Affiliations:** Department of Psychology, University of Bonn, Kaiser-Karl-Ring 9, 53111 Bonn, Germany; jschmuck@uni-bonn.de (J.S.); evoltz@uni-bonn.de (E.V.)

**Keywords:** opposite-gender bias, gender differences, social cognition, emotional faces, P1, N170

## Abstract

Studies of social cognition have shown gender differences regarding human face processing. One interesting finding is the enhanced processing of opposite-gender faces at different time stages, as revealed by event-related brain potentials. Crucially, from an evolutionary perspective, such a bias might interact with the emotional expression of the face. To investigate this, 100 participants (50 female, 50 male) completed an expression-detection task while their EEG was recorded. In three blocks, fearful, happy and neutral faces (female and male) were randomly presented, with participants instructed to respond to only one predefined target expression level in each block. Using linear mixed models, we observed both faster reaction times as well as larger P1 and late positive potential (LPP) amplitudes for women compared to men, supporting a generally greater female interest in faces. Highly interestingly, the analysis revealed an opposite-gender bias at P1 for happy target faces. This suggests that participants’ attentional templates may include more opposite-gender facial features when selectively attending to happy faces. While N170 was influenced by neither the face nor the participant gender, LPP was modulated by the face gender and specific combinations of the target status, face gender and expression, which is interpreted in the context of gender-emotion stereotypes. Future research should further investigate this expression and attention dependency of early opposite-gender biases.

## 1. Introduction

Humans are remarkably good at detecting and recognizing emotions in faces. This is not surprising given their importance to our ancestors, signaling either danger or opportunities, and therefore, guiding our behavior based on this identification. As early as 100 ms after stimulus appearance, people can direct their gaze toward faces [1] and rough affective categorization of emotional faces occurs [2]. Individual face recognition on a behavioral level appears to be feasible in just a quarter of a second [3], with humans generally performing very well in facial recognition tests [4]. Similarly to other fields of social cognition, differences between women and men have also been observed in facial processing [5]. For example, women have been shown to be better than men at remembering human faces [6,7] and identifying facial affect [8,9,10].

Moving beyond behavioral findings, gender differences can also be seen on the neurophysiological level. Here, the rapid neural processing of faces can be especially well-captured by event-related brain potentials (ERPs). Various ERP components at different time stages have been associated with face processing. At a very early stage, the visual P1 peaking around 100 ms at occipital electrodes and originating from extrastriate visual areas [11,12] already shows face-sensitivity; that is, it is greater for faces compared to other objects [13,14,15,16]. While the P1 effect is supposedly driven by low-level visual features [15,16], the robust face-sensitivity (faces > other stimuli) of the occipito-temporal N170 at around 170 ms likely reflects genuine configural face processing [17,18,19]. In contrast to the earlier P1, N170 is also reliably modulated by emotional facial expressions, with more negative (i.e., greater) amplitudes for emotional compared to neutral faces (for a review, see [20,21]). At later stages, the late positive potential (LPP) reflects controlled as well as automatic attentional processes elicited by motivationally significant stimuli [22,23,24]. It is a sustained positive deflection over centroparietal electrodes, which begins within 300 ms after stimulus onset [23]. The more elaborate stimulus processing at the LPP is evident in the sensitivity to the presence of human faces in neutral pictures [25,26,27], (the degree of) the affect of facial expressions [21,28], the attractiveness of faces [29,30] and the faces of romantic partners [31].

In line with the greater emotional perceptiveness and reactivity of women (see [8,10]), EEG studies reported enhanced activity in females compared to males when observing facial expressions. In particular, women typically show larger amplitudes of P1 [32,33,34,35,36] and the LPP [32,33,37,38]. The findings have been less consistent for the N170 (for the differences between genders, see [33,36,39], but also see [40]). The enhanced early visual as well as sustained attentional processing might be explained by the presumed greater evolutionary importance of social relations for women than men and their greater role in parental care [5,41]. Nevertheless, cultural influences and an interaction between both factors likely play a role (see [10,42]). However, the ERP amplitudes do not always differ between men and women (P1: [40]; N170: [32]) or are even larger for men than women (LPP: [43]). These divergent findings could result from differences in the experimental tasks, but it is also highly plausible that not all faces influence the neural processing in the observer equally [8].

For example, human faces convey important demographic information about gender, ethnicity and age, all of which were found to modulate the brain’s response to faces [44,45]. Focusing on face gender, electrophysiological studies did not find clear evidence of main effects on early visual components such as P1 or N170 ([44,46,47,48,49,50]; but see [51]), but rather on later components such as P200/N200 [44] and P300/LPP [43,49]. While the direction of the ERP effects differed between the P200 and N200 components [44], the LPP was increased for female faces compared to male faces [43,49]. Crucially, the effects of face gender may interact with the gender of the observer. From an evolutionary perspective [52], it could be reasoned that individuals generally pay more attention to potential mates; that is, men may attend more to women and vice versa. Using a recall paradigm, Hofmann et al. [53] indeed observed better recognition of the identity of opposite-gender faces. Correspondingly, research from fMRI studies revealed that female and male faces generally evoke similar neural activation within a distributed cortical network [54]. Interestingly, and in line with an evolutionary mating account, participants showed stronger neural activation within reward-related areas such as the nucleus accumbens or ventromedial prefrontal cortex during anticipation and viewing of opposite-gender compared to same-gender faces [54,55,56,57].

However, few EEG studies have tracked the time course of this opposite-gender bias in face processing. Employing a covert-orienting task, van Hooff et al. [30] found larger P2 amplitudes over parietal electrodes in response to opposite-gender faces, presumably reflecting early attentional capture by distinctive faces. Using an oddball task, S. Zhang et al. [58] observed differences between women and men in opposite-gender face-processing starting at around 200 ms. In both studies, however, no same-gender faces were presented, making it difficult to assess the difference to opposite-gender faces. In a gender discrimination task, Suyama et al. [59] observed a larger P2 component at temporal sites in men viewing female compared to male faces. Likewise, women showed enhanced processing of male versus female faces; this effect, however, was located over central sites at around 170 ms. Thus, the location and the timing of the opposite-gender bias differed between females and males. Finally, Proverbio et al. [60] provided additional evidence of an opposite-sex bias in face processing, which was reflected in a larger and earlier centro–parietal N400 for opposite-gender compared to same-gender faces when the faces were passively viewed. Interestingly, they also observed a greater LPP in response to same-gender faces than opposite-gender faces at occipitoparietal sites, which they suggested might be related to the representation of self-images. This is in line with the greater P300 [43] and LPP [49,61] amplitudes for same-gender faces compared to opposite-gender faces. However, this mainly applies to women, because men were either not included in the study [49] or there was simply no difference in men [43]. Summarizing the ERP findings, men and women seem to process opposite-gender faces earlier than same-sex faces, which are only more intensively processed at late components (see [60]).

However, from a mating perspective, not only could face gender be particularly attended to but (emotional) facial expressions could also provide critical cues during social interactions. A happy, smiling face indicates positive mood and liking, implying that it is worth approaching and interacting socially with someone who smiles at you [62,63]. Moreover, smiling (i.e., happy faces) was found to enhance both female and male facial attractiveness ([64,65,66,67]; but see [68]) and to be positively associated with trustworthiness [69,70]. Both trustworthiness and attractiveness have been identified as important criteria for mate selection [71,72], so an opposite-gender bias related to mating might be particularly evident in happy, smiling faces. Supporting the emotion-specificity, Conway et al. [73] reported a stronger attraction to direct gaze in happy faces than in disgusted faces, particularly for attractiveness judgments of opposite-gender faces. Emphasizing the mating perspective on the bias, electrophysiological findings revealed an opposite-gender bias in the contingent negative variation (CNV), an ERP marker of anticipatory attention, when participants viewed pictures of female and male nudes [74,75,76]. Apart from the described facial cues, such as happiness, body cues are also important in determining physical attractiveness [77] and might therefore elicit an opposite-gender bias in neural processing. Returning to facial stimuli, only one study has investigated opposite-gender biases related to different emotional expressions [61]. No opposite-gender bias was found, only an LPP enhancement for neutral (but not happy) expressions of same-gender faces compared to opposite-gender faces. However, limitations arise from the small sample size (*N* = 25), presumably lacking the power to find effects in the reported four-way ANOVA, and the fact that only two facial expressions (happy, neutral) were included. As it becomes obvious, there exists a lack of studies that track the time course of opposite- and same-gender biases depending on different emotional facial expressions [8].

The aim of the present study was an in-depth investigation of the electrophysiological indices of opposite-gender biases and their modulation by different facial expressions, both positive (i.e., happy) and negative (i.e., fearful). We built upon our previous study [78], which explored the processing of emotional facial expressions at different levels of attention using an expression-detection task. The results revealed early effects of expression (P1, N170) and suggested that selective attention toward a specific target expression promotes the processing of this very facial affect at early attentional stages [78]. More specifically, the early posterior negativity (EPN) arousal effect (emotional > neutral faces) was larger when the facial expression was selectively attended (i.e., a target) compared to when it was not (i.e., a nontarget). Given that this task induces selective attention to one specific facial expression, the task-irrelevant face gender will be processed non-intentionally (or automatically), which is similar to previous studies [30,60]. In order to investigate the conjoint effects of face gender and participant gender with sufficient power, we substantially increased the sample size of Schmuck et al. [78] up to *N* = 100, which is more than twice the number of participants in previous studies, such as those by Proverbio et al. [60] and van Hooff et al. [30], and matched the female and male participants in number. Our analysis focused on three components that have been shown to be sensitive to facial stimuli at different processing stages (e.g., [16,25]). These included the P1 as a marker of early visual processing, the N170 as a marker of holistic face encoding, and the LPP as reflecting late controlled processing and sustained attention toward stimuli.

Therefore, our hypotheses were as follows. First, on the behavioral level, it was shown that opposite-gender faces are recognized more quickly than same-gender faces, regardless of the facial expressions [53]. However, there is also evidence that specific combinations of expression and face gender (happy female and angry male faces) are identified particularly fast, irrespective of the participant gender [79]. Seeing these inconsistent previous results, no specific hypothesis for interactive effects between gender factors (face and participant) and facial expression on reaction times could be established. Second, building upon the current ERP literature, we assumed that the processing of opposite-gender faces would take place earlier than that of same-gender faces and would manifest itself in larger amplitudes toward opposite-gender faces (e.g., [30,60,61]). While the exact modulation of these effects by the three facial expressions should be explored in the present study, we expected an opposite-gender bias to be particularly apparent in happy (smiling) expressions due to their association with mate selection [71,80]. That is, when participants selectively attend to happy faces in the current task, they will pre-activate a rough visual representation of a typical happy face, the so-called attentional template (e.g., [81,82]). Since we assume that a typical happy face tends to be of the opposite gender (see above), these faces will better match the happy attentional template, and hence, opposite-gender faces should elicit larger amplitudes than same-gender faces. Third, in line with previous findings [32,36,37], we hypothesized that female (vs. male) participants would generally show larger ERP amplitudes to the faces, reflecting their greater interest in facial stimuli and the greater importance of social interactions for women.

## 2. Method

### 2.1. Participants

A total of 102 participants (*M_age_* = 23.8, SD_age_ = 3.8, range 19–42, 51 men, 51 women; all with normal or corrected-to-normal vision; no history of neurological or psychiatric disorders) took part in the current EEG study. The majority were recruited at the Department of Psychology at the University of Bonn and received partial course credit for their participation. This study was approved by the local ethics committee (#22-03-15) and all the participants provided written informed consent.

The final behavioral and EEG results include 100 participants (*M_age_* = 23.7, SD_age_ = 3.3, range 19–32, 50 men, 50 women). One participant had to be excluded due to technical problems during the EEG recording and one participant exhibited excessive noise in the EEG data (>40% rejected trials after channel interpolation).

### 2.2. Stimuli and Apparatus

**Figure 1:**
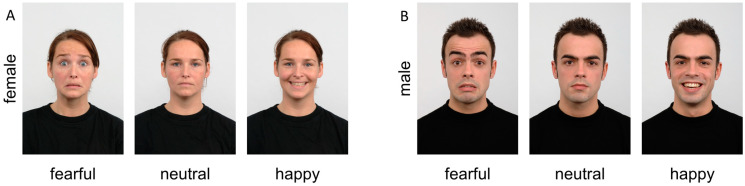
Sample images of female (**A**) and male (**B**) emotional faces from the RaFD (Langner et al., 2010 [83]).

We selected portraits of 38 (19 female and 19 male) different actors from the Radboud Faces Database (RaFD; Langner et al. [83]; see Figure 1 for sample faces; as the RaFD contains an unequal number of male (20) and female (19) Caucasian faces, we did not include the following male face: #Rafd090_21). The female and male actors did not differ regarding their attractiveness ratings reported in the RaFD, Welch’s *t*(35.74) = 1.50, *p* = 0.143. For each actor, we included frontal views of three facial emotional expressions (happiness, fear, neutral). This yielded a total of 114 different faces to be displayed as close-up color images (477 × 717 pixel). To investigate the potential arousal and valence differences between the three emotional expressions and the face gender, the mean ratings were obtained from the RaFD and each subjected to a two-way ANOVA.

For arousal, there was a significant effect of expression, *F*(2, 108) = 29.67, *p* < 0.001. Follow-up tests showed that fearful and happy faces had higher arousal ratings compared to neutral faces (all Bonferroni–Holm corrected *p*_c_ < 0.001), with no differences between the two sets of emotional expressions (*p*_c_ = 0.290). There was neither a main effect of the face gender, *F*(1, 108) = 0.87, *p* = 0.354, nor an interaction between the expression and the face gender, *F*(2, 108) = 1.27, *p* = 0.284. The valence ratings also differed significantly between the three expressions, *F*(2, 108) = 497.81, *p* < 0.001. The happy faces were rated more positively than the neutral faces, *p* < 0.001, which were in turn rated more positively rated than the fearful faces, *p* < 0.001. The female and male faces did not differ in their valence ratings, *F*(1, 108) = 0.52, *p* = 0.474, and there was no interaction between the expression and the face gender, *F*(2, 108) = 0.56, *p* = 0.575.

Furthermore, we analyzed the facial expressions and face gender for differences in the lower-level image features, including the mean brightness and mean contrast using ImageJ [84] and the size of the face (proportion of non-background pixels), mean proportion of the three color channels (red, green, blue), and median spectral frequency using additional scripts [85] running on MATLAB R2021a. All seven two-way ANOVAs (using each image feature as dependent and the expression and face gender as independent variables) showed neither an effect of expression, all *F*(2, 108) < 1.01, all *p* > 0.368, nor an interaction, all *F*(2, 108) < 0.06, all *p* > 0.940. The significant main effects of the face gender indicated that female and male face images only differed regarding the contrast (female > male), median spectral frequency (female < male) and the proportion of non-background pixels (female < male); for all three measures, 4.74 < *F*(1, 108) < 14.80, <0.001 < *p* < 0.032.

### 2.3. Procedure

Upon arrival, the EEG was prepared and the participants performed an expression-detection task with emotional faces. The task consisted of three blocks in which happy, fearful and neutral faces were randomly presented. In each block, the instructions were to press a button (space bar) as quickly and accurately as possible, but only in response to one specific (target) expression (happy, fearful or neutral) specified before each block. If the expression of the displayed face did not match the predefined target expression (nontarget trials), the response had to be withheld. The 114 faces were presented once per block in a random order, with the block order (and hence target expression) counterbalanced across participants. Between blocks, the participants could take a break if necessary.

At the beginning of each trial, a white fixation cross (40-point Arial font) was presented in the center of the screen for a random duration between 1000 and 1500 ms. It was followed by a face that disappeared on target trials when a response was made. In the absence of a response within 1500 ms, the feedback reading “Too slow!” was presented for 1000 ms. On nontarget trials, responses within 1500 ms prompted the feedback “Wrong!” which was shown for 1000 ms. All the stimuli were presented on a black background at the center of a 23″ TFT screen, with a resolution of 1920 × 1080, at a viewing distance of approximately 60 cm. The experiment was programmed and run on Presentation 21.1 (Neurobehavioral Systems Inc., Berkeley, CA, USA). After the EEG recording, all the participants answered sociodemographic questions and completed various personality trait measures, which were unrelated to the current study.

### 2.4. EEG Recording and Pre-Processing

The EEG was recorded from 61 Ag/AgCl electrodes placed according to the 10% system, using a digital 64-channel BrainAmp system (Brain Products, Gilching, Germany). The ground electrode was located close to AFz; FCz served as the reference electrode during the recording. The vertical and horizontal electrooculograms (EOG) were monitored from electrodes mounted below and above the right eye, and at the outer canthi, respectively. The EEG was recorded at a sampling rate of 500 Hz, and the impedances of all the electrodes were kept below 5 kΩ. The EEG data processing was performed using the Python module MNE [86], version 1.5.1, running on Python version 3.10.13. First, the continuous data were high-pass filtered at 0.1 Hz (Butterworth IIR filter with order 4) and low-pass filtered at 30 Hz (order 4). Additionally, a notch filter was applied at 50 Hz. Bad channels were automatically detected using the Python module pyprep, which implements the recommendations from the PREP Pipeline [87]. All the channels that deviated in terms of either the bad-by-high-frequency-noise or the bad-by-deviation criteria (with z score > 5) were interpolated using a spline interpolation procedure (up to 7 channels; mean = 2.01). The online reference electrode (FCz) was added as an empty channel and the data were subsequently re-referenced to the average reference. To correct for eye-blink artifacts, an ICA (*picard* algorithm; [88]) was conducted on a separate dataset, which was filtered with a high-pass filter at 1 Hz and a low-pass filter at 30 Hz [89] but otherwise identically pre-processed. The number of independent components (ICs) to be calculated was adjusted to match the rank deficiency of the data resulting from the earlier channel interpolation and average referencing [90]. We used MNE’s standard algorithm to detect problematic EOG data and to automatically identify ICs associated with ocular artifacts (as indicated by high correlations with ocular channels), using a threshold of z > 4. This resulted in the removal of 2.18 ICs on average. The ICA decomposition was then applied to the original dataset.

For the ERP analysis, continuous data were segmented into epochs of 1100 ms length (–100; 1000 ms) relative to the face onset and baseline corrected to the 100 ms pre-stimulus interval. Epochs containing amplitudes exceeding ±100 μV were rejected. Only those trials were included in the analyses on which the participants responded correctly as per the instructions. On average, 333.00 artifact- and error-free epochs (97.37% of all trials) were retained. The number of rejected trials due to EEG artifacts was not affected by the different experimental factors, all *F* < 2.11, *p* > 0.127, except for one interaction between expression, participant gender and face gender, *F*(2, 196) = 3.08, *p* = 0.048, for which, however, post hoc tests did not reveal significant differences for orthogonal comparisons between cells.

For our analyses, we focused on two early perceptual ERPs; that is, modulations of visual P1 and N170, and the LPP, reflecting more controlled stimuli processing. The time windows and areas of interest for these components were based on the grand–grand average ERP during face processing, collapsed across all the participants and experimental conditions. The parieto-occipital P1 peaked at 120 ms after stimulus onset. We therefore quantified the P1 amplitude in a symmetrical 30 ms time window (105–135) around the peak at two lateral clusters (left: PO3, PO7, O1; right: PO4, PO8, O2). The mean amplitude of N170, peaking at 160 ms, was measured between 145 and 175 ms at symmetrical occipital clusters (left: P7, PO7; right: P8, PO8). LPP was measured in a 300 ms time window (300–600 ms) at parietal electrodes (P3, P1, Pz, P2, P4, PO3, POz, PO4). The mean ERP amplitudes at the above-determined latency and location were extracted from the individual trial level per participant and then subjected to single-trial linear mixed effects (LME) analyses.

### 2.5. Statistical Analyses

All the computations were performed in R 4.3.0 (R Core Team, 2021). The complete research design included the experimental factors *status* (target and nontarget), (*facial*) *expression* (happy, fearful and neutral), *face gender* (female and male) and additionally the (*participant*) *gender* (female and male). For all the dependent behavioral and EEG variables, we computed the LME models using the function *mixed* from the package *afex* [91] and included all four factors as well as their interactions as fixed effects (except for RT; only target trials with RTs slower than 100 ms were considered since the participants should not respond in nontarget trials). For random effects, we employed the maximal structure in terms of the by-participant and by-stimuli intercepts, slopes and correlations that converged for each measure [92] and report it in the Results section. All the models were run using the *bobyqa* optimizer and Kenward–Roger approximation was applied to estimate the degrees of freedom and to obtain *p*-values from the main model. Significant interactions were followed up by running the models with factors forming the interaction within each other to obtain estimates for the comparison within each level of the respective other factor using the function *joint_tests* from the package *emmeans* [93]. Finally, in case of significant two-way interactions, two Bonferroni–Holm corrected *t*-tests using the function *emmeans* served to pairwise compare the difference in one factor at the levels of the other factors. The degrees of freedom and *p*-values for the *t*-tests were calculated using Satterthwaite’s method [94]. In all the analyses, we used *p*-values of 0.05 as a significance criterion unless noted otherwise. Importantly, to avoid overlaps with our previous study [78] and to focus on our current research question, only the main effects or interactions involving the *participant gender* and/or *face gender* are reported in the following. The complete results table are provided in Tables S1–S3 of the Supplementary Materials.

## 3. Results

### 3.1. Sociodemographic Differences

Regarding the sociodemographic variables, the independent two-sample Welch *t*-tests showed that the men were significantly older than the women, *M*_diff_ = 2.84, *SE* = 0.61, *t* = 4.69, *p* < 0.001, 95% CI [1.64, 4.04]. However, there was no association between gender and education, χ^2^(1) = 1.06, *p* = 0.303, gender and current job status, χ^2^(3) = 1.49, *p* = 0.685, or gender and handedness, χ^2^(2) = 1.30, *p* = 0.522.

### 3.2. Behavioral Results

The best-fitting LME model for the hit RT [RT ~ Gender × Face Gender × Expression + (Expression|Participant) + (1|Stimuli)] revealed a main effect of gender, *F*(1, 98) = 6.80, *p* = 0.011. Women responded significantly faster than men, *M*_diff_ = 41.23, *SE* = 15.81, *t* = 2.61, *p* = 0.011, 95% CI [9.85, 72.61]. In addition, the interaction between the face gender and the expression yielded a significant effect, *F*(2, 10,865) = 4.61, *p* = 0.010. The responses tended to be faster for female than for male faces when displaying a happy expression, *M*_diff_ = 15.42, *SE* = 6.91, *t* = 2.23, *p*_c_ = 0.088, 95% CI [–1.58, 32.41], but not when the faces were neutral or fearful, all *t* < 1.13, *p*_c_ > 0.529. All the other effects, including participant or face gender, remained not significant, all *F* < 2.59, *p* > 0.075.

Regarding the error rates, the best-fitting LME model [Error Rate ~ Status × Gender × Face Gender × Expression + (Expression|Participant) + (Expression|Stimuli)] yielded a main effect of status, *F*(1, 22,381) = 16.86, *p* < 0.001. There were significantly less errors in the nontarget trials (i.e., *false alarms*) compared to the target trials (i.e., *misses*), *M*_diff_ = 0.57, *SE* = 0.14, *t* = 4.11, *p* < 0.001, 95% CI [0.30, 0.84]. Therefore, we investigated the false alarms and misses separately. For the false alarms, we did not find any significant effects involving gender or face gender, all *F* < 0.35, *p* > 0.558. However, focusing on the misses, we observed a main effect of gender, *F*(1, Inf) = 6.06, *p* = 0.015. Overall, women committed significantly less errors of omission than men, *M*_diff_ = 0.74, *SE* = 0.30, *t* = 2.46, *p* = 0.015, 95% CI [0.15, 1.33]. Additionally, there was a two-way interaction between gender and face gender, *F*(1, Inf) = 11.71, *p* < 0.001, as well as gender and expression, *F*(2, Inf) = 4.30, *p* = 0.014. First, women made less misses compared to men when female faces were presented, *M*_diff_ = 1.40, *SE* = 0.36, *t* = 3.93, *p*_c_ < 0.001, 95% CI [0.60, 2.21], but not regarding male faces, *M*_diff_ = 0.07, *SE* = 0.36, *t* = 0.20, *p*_c_ = 0.844, 95% CI [–0.73, 0.87]. Second, the overall pattern (women < men) was particularly evident in the fearful faces, *M*_diff_ = 1.53, *SE* = 0.42, *t* = 3.67, *p*_c_ < 0.001, 95% CI [0.53, 2.53], but it did neither emerge in the neutral or happy faces, all *t* < 1.37, *p*_c_ > 0.345.

### 3.3. EEG Results

First, to investigate the potential differences in hemisphere processing, we calculated the LME models including *laterality* (left, right) for both P1 and N170. For P1, there was only a main effect of laterality (right > left), *F*(1, 66,223) = 35.72, *p* < 0.001. For N170, there was again a main effect of laterality, *F*(1, 66,125) = 140.39, *p* < 0.001, as well as two interaction effects involving laterality, both *F* > 3.27, *p* < 0.038. The amplitude was more negative (i.e., greater) on the right compared to the left hemisphere. However, as there were few interaction effects of hemisphere with our factors of interest, we only report the results for the respective LME models averaged across the hemispheres.

#### 3.3.1. P1 (105–135 ms)

**Figure 2:**
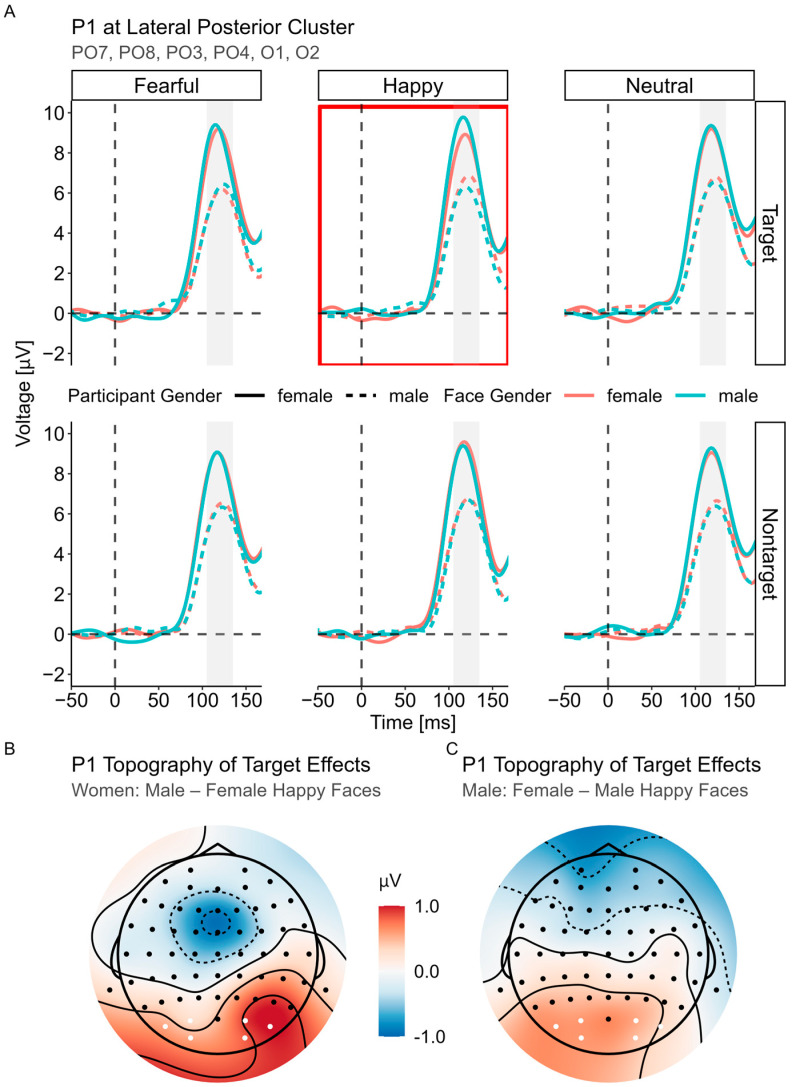
P1 component. (**A**) Averaged ERPs for participant gender and face gender are shown separated by status and expression. The red rectangle highlights the source of the largest interaction effect, which is the significant two-way interaction between gender and face gender for the happy target faces. (**B**,**C**) Difference topography maps for the significant pairwise comparisons of the two-way interaction.

The best-fitting LME model was P1 ~ Status × Gender × Face Gender × Expression + (Face Gender |Participant) + (1|Stimuli). It yielded a main effect of gender, *F*(1, 98) = 11.04, *p* = 0.001, indicating that women had a larger P1 amplitude than men, *M*_diff_ = 2.36, *SE* = 0.71, *t* = 3.32, *p* = 0.001, 95% CI [0.95, 3.76]. Interestingly, there was a significant four-way interaction between expression, status, gender and face gender, *F*(2, 33,055) = 4.49, *p* = 0.011. To follow up on this effect, we analyzed the three-way interaction at each level of expression. This revealed a highly significant interaction only for the happy faces, *F*(1, Inf) = 9.71, *p* = 0.002, not for the other expressions, both *F*(1, Inf) < 0.72, *p* > 0.400. Focusing on the happy faces, scrutiny of the gender × face gender interaction at the two levels of status showed a significant interaction arising from the target faces, *F*(1, Inf) = 9.53, *p* = 0.002, but not from the nontargets, *F*(1, Inf) = 0.96, *p* = 0.327. Finally, on the 2 (gender) × 2 (face gender) level, the post hoc *t*-tests indicated that women had larger P1 amplitudes in response to male (vs. female) happy target faces, *M*_diff_ = 0.64, *SE* = 0.28, *t* = 2.27, *p*_c_ = 0.047, 95% CI [0.01, 1.26], while men had larger P1 amplitudes in response to female (vs. male) happy target faces, *M*_diff_ = 0.57, *SE* = 0.28, *t* = 2.03, *p*_c_ = 0.047, 95% CI [0.06, 1.20]. That is, we observed an opposite-gender bias specific to happy target faces in the P1 time range (see Figure 2). Further effects involving gender and/or face gender were not significant.

Given that we found differences between women and men in terms of age, we additionally controlled for (standardized) age in a separate P1 LME model to better isolate the gender-specific effects. This did not change the significance of the four-way interaction, *F*(2, 33,055) = 4.49, *p* = 0.011. However, the main effect of gender did not reach statistical significance anymore, *F*(1, 98) = 3.43, *p* = 0.067, in contrast to the participants’ age, *F*(1, 97) = 8.84, *p* = 0.004. Here, the P1 amplitude decreased with age, *b* = −1.12, *SE* = 0.38.

#### 3.3.2. N170 (145–175 ms)

**Figure 3:**
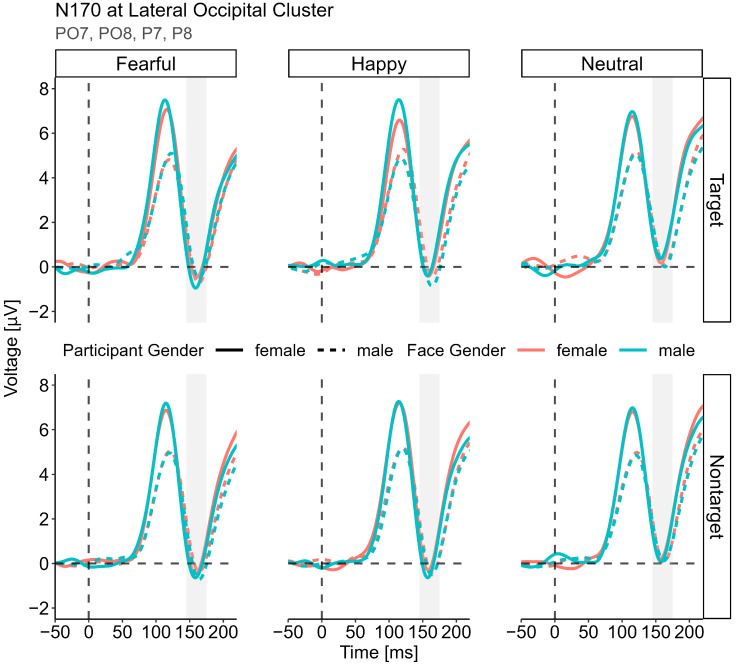
N170 component. Averaged ERPs for participant gender and face gender are shown separated by status and expression. In contrast to P1, no significant two-way interactions between gender and face gender emerged at the different status and expression levels and therefore no topography maps for any pairwise comparisons are shown.

The best fitting LME model for the N170 data [N170 ~ Status × Gender × Face Gender × Expression + (Face Gender + Expression|Participant) + (1|Stimuli)] only revealed a significant four-way interaction, *F*(2, 32,880) = 3.27, *p* = 0.038 (apart from the typical N170 effect of expression; emotional < neutral). The interaction was best investigated by comparing the three-way interaction between expression, gender and face gender at the two levels of status (see Figure 3). While the interaction was significant for the target faces, *F*(2, Inf) = 3.24, *p* = 0.039, this was not the case for the nontarget faces, *F*(2, Inf) = 0.34, *p* = 0.710. Among the target faces, the follow-up analysis revealed a trend toward a significant gender × face gender interaction in the happy faces, *F*(1, Inf) = 3.31, *p* = 0.069, and fearful, *F*(1, Inf) = 3.00, *p* = 0.083, but not in the neutral faces, *F*(1, Inf) = 0.23, *p* = 0.635. None of the follow-up *t*-tests on the 2 × 2 level (female vs. male faces by participant gender) reached significance, neither among the happy faces (all *t* < 2.03, *p*_c_ > 0.085) nor the fearful faces (all *t* < 1.45, *p*_c_ > 0.292).

Due to the spatial and temporal proximity of the N170 to the P1 amplitude, we analyzed a second LME model including the P1 amplitude as a nuisance variable. The main effect of expression on the N170 still remained highly significant, *F*(2, 136) = 43.28, *p* < 0.001; however, and importantly, the four-way interaction no longer was, *F*(2, 32,869) = 1.59, *p* = 0.204. Moreover, the three-way interactions separated by status did not yield any significance, all *F* < 1.34, *p* > 0.262, as well as all the lower-order gender × face gender interactions reported above, all *F* < 2.34, *p* > 0.126.

#### 3.3.3. LPP (300–600 ms)

Significant main effects of both face gender, *F*(1, 42) = 8.33, *p* = 0.006, and gender, *F*(1, 98) = 12.48, *p* < 0.001, emerged from the best fitting LME model [LPP ~ Status × Gender × Face Gender × Expression + (Status + Expression|Participant) + (1|Stimuli)]. The LPP amplitude was significantly larger for female (vs. male) participants, *M*_diff_ = 1.77, *SE* = 0.50, *t* = 3.53, *p* < 0.001, 95% CI [0.78, 2.77]. Additionally, female faces elicited a greater LPP compared to male faces, *M*_diff_ = 0.21, *SE* = 0.07, *t* = 2.89, *p* = 0.006, 95% CI [0.06, 0.36]. Following up on the significant *status* × *gender* interaction, *F*(1, 98) = 12.06, *p* < 0.001, revealed that the LPP difference between both genders (female > male) was larger in the target condition, *M*_diff_ = 2.25, *SE* = 0.58, *t* = 3.91, *p*_c_ < 0.001, 95% CI [0.94, 3.57], than in the nontarget condition, *M*_diff_ = 1.29, *SE* = 0.58, *t* = 2.83, *p*_c_ = 0.005, 95% CI [0.25, 2.33].

**Figure 4:**
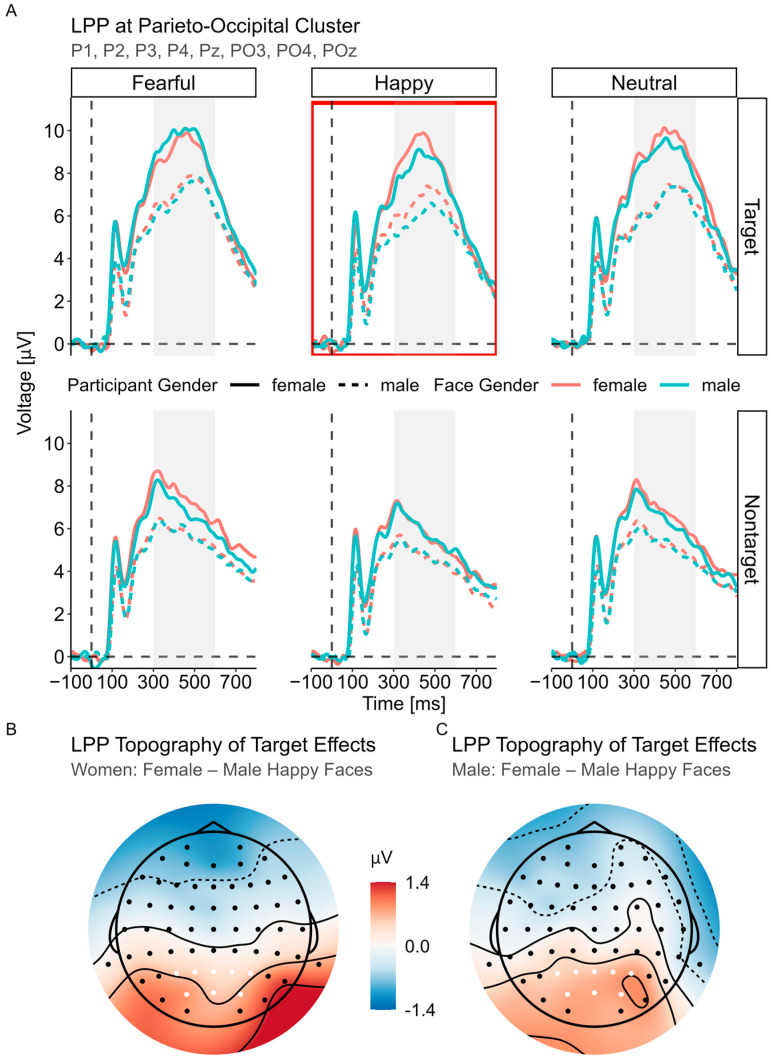
LPP component. (**A**) Averaged ERPs for participant gender and face gender are shown separated by status and expression. The red rectangle highlights the source of the largest interaction effect, which is the significant two-way interaction between expression and face gender for the target faces. (**B**,**C**) Difference topography maps for the significant pairwise comparisons between female and male happy target faces.

There was a significant three-way interaction between status, expression and face gender, *F*(2, 32,862) = 6.44, *p* = 0.002 (see Figure 4). Scrutiny of this interaction revealed a highly significant expression × face gender interaction in the target faces, *F*(2, Inf) = 4.68, *p* = 0.009, compared to the nontarget faces, *F*(2, Inf) = 1.97, *p* = 0.140. Among the target faces, female (vs. male) faces elicited a larger LPP only when they had a happy expression, *M*_diff_ = 0.63, *SE* = 0.17, *t* = 3.62, *p*_c_ < 0.001, 95% CI [0.21, 1.05], not when the expression was neutral, *M*_diff_ = 0.27, *SE* = 0.18, *t* = 1.52, *p*_c_ = 0.258, 95% CI [–0.15, 0.69], or fearful, *M*_diff_ = –0.10, *SE* = 0.17, *t* = –0.56, *p*_c_ = 0.573, 95% CI [–0.52, 0.32]. These differences did not emerge in the nontarget faces, all *t* < 2.22, *p*_c_ > 0.081.

## 4. Discussion

The current ERP study investigated the differences in the neural processing of opposite- and same-gender faces and the extent to which these are influenced by the emotional expression of the faces. While previous studies primarily studied a single facial expression [30,58,60], no study has used both positive (here: happy), negative (here: fearful) and neutral expressions. In each block, the participants had to respond to one of the three specific (target) expressions, which was specified before each block, and to withhold their responses to the other two expressions. Each expression was therefore either selectively attended (i.e., a target) or non-attended (i.e., a nontarget), while in contrast, face gender was processed non-intentionally (or automatically) in all the trials.

This is similar to previous ERP studies investigating the opposite-gender bias (e.g., [30,58,60,61]), which also focused on the automatic processing of face gender. Indeed, we found evidence of an early opposite-gender bias at the visual P1 in our expression detection task. Importantly and in line with our theoretical considerations, this effect only emerged in happy (target) faces, not in the other expressions. Specifically, the P1 for women was greater for male (vs. female) happy target faces, and for men, the P1 was greater for female (vs. male) happy target faces. Regarding N170, there was no strong indication of any bias toward opposite- or same-gender faces. During the LPP time window, we again did not observe any interaction between face gender and participant gender; however, happy female (target) faces generally elicited a greater LPP amplitude than happy male faces. Finally, the main effects of participant gender yielding greater amplitudes for women compared to men emerged at both early and late stages (e.g., [32,33]), while face gender only modulated the LPP amplitude (e.g., [43,49]). The main effects of expression were also revealed at all three components and at the behavioral level; however, these, as well as the LPP effects of status, are discussed in more detail in Schmuck et al. [78], while the current study focused on effects involving participant and/or face gender.

The behavioral findings indicated that women were both faster and made less errors of omission in the current task compared to men. Even though similar previous studies (e.g., [30,58,60,61]) did not report RTs or error rates, our current results can be integrated into the larger body of literature on emotion processing [8] and gender-emotion stereotypes [95,96]. The female advantage is well in line with a better ability of women to recognize facial emotional expressions in a variety of emotion recognition tasks (for reviews, see [8,97]). This might relate to the evolutionary role of women as the primary caretaker of the offspring and hence the need to faster identify facial expressions as well as specific differences regarding socialization [5,9,10]. Furthermore, we observed an interaction effect for RTs between facial expression and face gender. There was no indication of any opposite- or same-gender bias, but rather specific combinations of facial features, which influenced the behavioral responses. In particular, only for the happy expressions, the participants tended to react faster to female faces than to male faces. This finding fits well with an implicit association between female faces and happy expressions [79,98]. There is also evidence of an association between male and angry; this could not be investigated in the current study as fearful rather than angry faces were used as negative stimuli. Descriptively, however, the above RT pattern (female < male) also emerged for fearful faces, which would be in line with Hess et al. [98]. The lack of any opposite-gender bias as was found, for example, in Hofmann et al. [53] might be explained by the task requirements. While the former study revealed faster naming of opposite-gender faces in a recall task, the current task creates a strong focus on the emotional expressions. Here, the somewhat “congruent” facial features (happy/female) compared to the “incongruent” facial features (happy/male) might have facilitated the detection of happy expressions, while the mere facial features of the opposite sex were not directly helpful for rapid behavioral expression detection.

The main goal of the current study was to investigate the ERP markers of an opposite- or same-gender bias in neural processing. Hence, we focused on three components that have shown sensitivity to faces, namely P1, N170 and LPP (e.g., [16,25]). For P1, women had significantly higher amplitudes than men, which is supported by various studies (e.g., [32,35,36]). Considering that P1 is an early marker of visual attention [11] and might even be sensitive to the motivational salience of faces [99], the finding of enhanced sensory processing in women (vs. men) could arise from a greater female interest in human faces as well as the supposed greater evolutionary importance of social relations for women than men [5,41]. There was no modulation by emotional expressions, suggesting that the initial processing advantage of women applies to all the expressions similarly, at least the ones used in the current study. It should be noted, however, that the gender effect was mitigated when controlling for the participants’ age due to existing age differences between men and women. While women still tended to have larger amplitudes than men, the P1 amplitude also significantly decreased with age (see [100,101]).

Importantly, we observed an early opposite-gender bias during the P1 time window. As hypothesized, this particular effect only emerged in happy faces, as it was found in neither fearful nor angry faces. Even more interesting is the fact that the effect was restricted to the target faces and was not present in the nontarget faces, as could be seen in the significant four-way interaction. In other words, only when the participants selectively attended to happy faces and a happy face actually appeared, opposite-gender faces elicited a higher P1 amplitude in the participants than same-gender faces; that is, male (vs. female) faces elicited a higher P1 in women, while female (vs. male) faces elicited a higher P1 in men. This finding of an attention- and expression-dependent opposite-gender bias for faces is highly interesting and will be discussed in the following. Previously, using an analogous task with emotional words, Gibbons et al. [102] found greater P1 emotion discrimination for targets compared to nontargets, suggesting that selective attention to affective stimuli can modulate the early P1 component through the creation of target templates (a central concept of feature-based attention; see [81,103]). That is, participants tune to specific features of the target stimuli, which might facilitate rapid integration and perceptual discrimination of presented target stimuli, thus eliciting early P1 effects. Building on this idea, we propose that participants in the attend-to-happy blocks preactivated neural representations of happy faces (for attentional tuning to faces, see [82]). Crucially, from an evolutionary perspective, searching for happy faces that convey more trustworthiness [69] and attractiveness [66] than other facial expressions also includes a notion of mate selection. Thus, among the female participants, the attentional templates of happy faces might include more male-related facial features, while the male participants might emphasize more female-related facial features in their template for happy faces. These features of the attentional templates might not even be intentionally created but can also be automatically represented as related to evolutionary constraints. This results in a particular early and strong match for happy female target faces in men and happy male target faces in women, eliciting an increase in the P1 amplitude (for P1 effects in relation to target stimuli, see also [104]). In contrast, the nontarget happy faces, irrespective of the face gender, always mismatched the attentional template (of neutral or fearful faces), thus revealing no P1 differences. Such specificity for the target faces emphasizes the notion that top-down tuning and attentional templates are crucial for the early P1 opposite-gender bias, given that P1 indexes selective and also feature-based attention [11,105] but does not reflect more complex interactions between bottom-up and top-down processes emerging at later processing stages. The absence of the target-sensitive opposite-gender effect in the other expressions might be related to the clear and distinct features of happy faces (such as a smile), which are particularly easy to represent in both female and male target templates, and/or to the fact that neutral and fearful expressions may be unrelated to mating.

Previous studies might not have revealed any P1 opposite-gender bias because they either did not use happy faces [30,58] or investigated smiling faces under passive viewing conditions [60]. Here, we propose that selective attention and the representation of target templates is necessary to elicit a very early opposite-gender bias in happy faces. As a caveat, we have to add that the female and male faces in the current experiment differed slightly regarding the lower-level image features to which the P1 component is highly sensitive [16]. However, these differences cannot explain the above results as the direction of the P1 effect was reversed in men and women and no differences between the same female and male faces emerged in the nontarget condition. Additionally, even though men and women differed regarding age, including age as a fixed effect did not alter the statistical significance of this interaction, which emphasizes the importance of participant gender and face gender combined.

Following P1, we also observed a significant four-way interaction during the N170 time window. In sharp contrast to the P1 analysis, this effect could not be conclusively elucidated as all the two-way interactions of participant and face gender at the different levels of expression and status turned out to be non-significant. Since the preceding P1 reached its maximum shortly before the N170 at similar parieto-occipital regions and showed large differences between men and women, which might have influenced the absolute voltage of the subsequent N170, we additionally controlled for the P1 amplitude in the respective linear mixed model. This revealed that the four-way interaction was no longer significant while all the other effects remained qualitatively unchanged. Furthermore, there were no interactions between face gender and specific expressions during this time window (as reported for angry faces by Valdes-Conroy et al. [49]) and also no effect of participant gender ([33,40,106,107]; but see [39]). In the current study, the only robust modulation of the N170 was driven by the emotional expression, showing the typical pattern of larger (i.e., more negative) amplitudes for happy and fearful faces compared to neutral faces (see Supplements and [78]; for a review, see [20]). Therefore, even though at earlier time stages selective attention modulated the processing of happy opposite-gender faces, the configural face processing [18] was influenced by neither task nor face gender. This is consistent with the idea that the N170 for faces in particular shows a very reliable effect of emotional expression that is stable across various attentional conditions ([21]; for the current task, see also [78]).

Finally, the LPP was modulated by participant gender, with significantly enhanced amplitudes for women compared to men. This is in line with various studies on emotional faces [32,33,37,38,108,109], suggesting that human faces might have greater motivational significance for women and thus attract more attentional resources during late processing stages. The specificity of this effect of social stimuli (faces or persons) is supported by the larger ERP responses to social stimuli vs. (emotional) scenes for women compared to men (N2: [110]; LPP: [111]). Nevertheless, it is conceivable that women were also more engaged (cf. [112]) and allocated more attentional resources to the current task. Correspondingly, we observed an interaction between gender and status, which revealed that women in particular showed a larger LPP increase for target faces compared to nontarget faces. Using different stimuli, previous studies also observed that women but not men had particularly enhanced amplitudes for task-relevant stimuli during late processing stages [113,114]. Therefore, probably due to both the greater motivational significance of human faces and the allocation of more attention to task-relevant stimuli, women had a greater LPP amplitude than men.

Furthermore, a main effect of face gender emerged during the LPP time window, yielding a larger LPP for female than male faces. This finding corroborates that a reliable discrimination of face gender independently of participant gender only appears at later processing stages [44,48] and is consistent with previous results that proposed a deeper or more detailed processing of female (vs. male) faces [43,49]. One possible explanation for this pattern could be that women express their emotions more often through facial expressions, while men hold their emotions to themselves or express themselves relatively more often through their non-mimic behavior [115,116]. Thus, female faces likely convey more relevant social information and need to be more strongly attended. Interestingly, there was an interaction between face gender, status and expression, which was again driven by the happy target faces. Here, female happy target faces had larger LPP amplitudes than male happy target faces. These differences between female and male faces were observed neither for fearful nor neutral target expressions and did also not emerge in any of the nontarget facial expressions. In contrast to P1, this effect was independent of participant gender, thus excluding any opposite- or same-gender bias. It is widely assumed that the LPP functions as a measure of motivational and emotional salience [117]. In line with our behavioral findings, we suggest that the congruency of expression and gender information in happy female faces [95] emphasized the emotional salience such that its LPP effects were larger compared to happy male faces. Of note, this enhancement was particularly evident when the happy faces were task-relevant and greater attention was allocated to their detailed processing. However, as nontargets, both the male and female happy faces do not match the target expression (are thus “incompatible”) and therefore both equally do not require greater processing. Similarly, previous studies found larger LPP amplitudes for stimuli matching gender-emotion stereotypes [118] and emotionally congruent stimuli not including face gender [119,120,121]; however, enhanced LPP amplitudes were also found when the facial expressions and context were incongruent [122,123].

Having discussed the behavioral and ERP findings in detail, we will briefly integrate them into an existing theoretical framework. The differences between females and males can develop when individuals are exposed to sex-specific evolutionary pressure, which is often due to sexual selection [96]. From the evolutionary perspective, mating preferences might therefore underlie our P1 opposite-gender bias, as it was specifically observed in relation to happy faces, which are strongly related to mate selection [80]. From this perspective, the evolutionary role of women as the primary caretakers can also explain their enhanced ERP amplitudes [5]. Additionally, the (face) gender-emotion interactions from both the behavioral and ERP findings have previously been related to the co-evolution of the presence of sex signals and specific emotional expressions [95,96]. However, our results are likely not only attributable to purely evolutionary/biological factors; social and cognitive factors probably also play a role. Here, differences in (emotional) socialization [9] could contribute to the enhanced processing of facial stimuli in women and an overlap between stereotypes or evaluative associations regarding gender and certain emotional expression could explain the observed gender-emotion interactions [96].

Finally, a few limitations should be noted with regard to our study. First, our sample consisted mostly of undergraduate psychology students. However, the participants’ gender was balanced and comprised 50 females and 50 males. This is more than double the sample size of previous ERP studies on opposite-gender bias [30,60,61], increasing the robustness of the results. We must note that sexual orientation was not initially assessed, so information was only available for part of the sample (*n* = 46; we re-analyzed the opposite-gender bias at P1, excluding all the participants who stated that they did not identify as heterosexual (5 men, 6 women). The four-way interaction and the lower-order interactions in the LME model remained significant and the pairwise comparisons even tended to show more pronounced differences (women: male > female happy target faces, *M*_diff_ = 0.70, *SE* = 0.30, men: female > male happy target faces, *M*_diff_ = 0.60, *SE* = 0.30). Therefore, we would cautiously assume that our results and conclusions are also correct for a purely heterosexual sample). This should be more clearly differentiated in future studies investigating expression-dependent opposite-gender biases. Second, the two groups significantly differed in terms of their age, which was previously found to have an effect on various ERP components, including P1 [100]. While both groups were not matched regarding age, we statistically controlled for age in our analysis. For P1, this indeed altered the main effect of gender, but it had no effect on the opposite-gender bias. Nevertheless, women and men might still differ in some confounding variables (other than education and handedness, which did not differ) that were not taken into account but may have influenced the ERP components. Third, the images of female and male faces varied regarding low-level image features such as the contrast and median spatial frequency. In particular, these could have accounted for the early ERP effects of face gender; however, it is implausible that they contributed to the opposite-gender bias, given the reverse P1 pattern in men and women, and its target dependency. Additionally, we used linear mixed models to consider these potential effects of the individual facial stimuli in our analysis. Among all the facial stimuli, we included fearful expressions as negative stimuli because they matched happy faces regarding arousal ratings. Here, one particularly interesting question for the future might be if and how angry faces would affect the processing of opposite- or same-gender faces. It is conceivable that angry (male) faces might be more threatening for men than for women (see [8]), thus they might elicit a same-gender bias in neural processing. Furthermore, the LPP pattern might be the reverse of the one in happy target faces, with angry male faces having larger amplitudes than angry female faces (see [118]). More generally speaking, opposite- and same gender biases might emerge only in certain emotional expressions, as was observed by Doi et al. [61] and in the current study.

## 5. Conclusions

To conclude, in the present expression detection task, we observed larger P1 and LPP amplitudes for females compared to males, which is most likely associated with the greater interest in and significance of human faces for women. Highly interesting was the finding of an early visual opposite-gender bias during the P1 time range, which was restricted to the target faces. In line with an evolutionary perspective of rapid attraction by potential mating partners, this bias was only found in happy faces and did not emerge in fearful or neutral faces. That is, when anticipating happy faces, men rather seem to picture female happy faces, while women rather imagine male happy faces. While the emotion effect of face/N170 was robust to the attentional condition as well as the participant/face gender, only the LPP showed a modulation by face gender. Female faces were generally processed more deeply at late controlled stages, while happy female target faces in particular attracted most attentional resources. This LPP finding as well as the behavioral findings likely reflect specific underlying gender-emotion stereotypes [95], which increased the emotional significance of the presented faces.

## Data Availability

The dataset is available on request from the authors due to ethical restrictions.

## Supplementary Materials

The following supporting information can be downloaded from the https://www.mdpi.com/article/10.3390/brainsci14080739/s1: Table S1: Linear mixed model for P1; Table S2: Linear mixed model for N170; Table S3: Linear mixed model for LPP.
