# Supplementary material for: You’re Beautiful When You Smile: Event-Related Brain Potential (ERP) Evidence of Early Opposite-Gender Bias in Happy Faces
Source: Brain Sci. 2024 Jul 24;14(8):739. doi: 10.3390/brainsci14080739 (PMC11353154; doi:10.3390/brainsci14080739)
Supplement: Supplementary file 1 [file brainsci-14-00739-s001.zip › supplementary_materials.pdf]

Supplementary Materials

In the following, we report the complete result tables for all three linear mixed models which we computed in our analysis. In Table S1, the complete results for the P1 amplitude can be found and in Table S2 and S3, the results for the N170 and the LPP are shown, respectively. Additionally, we followed up all significant effects of *Status* and/or *Expression* in order to give a comprehensive overview. All other significant effects (those involving participant and face gender) are already reported in the results section of the article.

Table S1.

Linear Mixed Model for P1 (105 – 135 ms).

Effect	df, df_{error}	*F*	*p*
Status	1, 33050.63	0.04	.847
Expression	2, 33053.64	3.95	.019
Gender (Face)	1, 41.11	0.46	.503
Gender	1, 98.21	11.04	<.001
Status × Expression	2, 33051.63	1.11	.330
Status × Gender (Face)	1, 33054.78	1.58	.209
Expression × Gender (Face)	2, 33055.53	0.11	.897
Status × Gender	1, 33049.83	0.55	.458
Expression × Gender	2, 33050.24	0.01	.994
Gender (Face) × Gender	1, 121.46	2.65	.106
Status × Expression × Gender (Face)	2, 33055.49	0.21	.808
Status × Expression × Gender	2, 33050.71	0.70	.496
Status × Gender (Face) × Gender	1, 33053.97	1.45	.229
Expression × Gender (Face) × Gender	2, 33052.14	1.74	.175
Status × Expression × Gender (Face) × Gender	2, 33054.57	4.49	.011

For P1, there was a significant main effect of *Expression*, $F(2,33054) = 3.95$, $p = .019$. Happy faces elicited a larger P1 amplitude compared to fearful faces, $M_{diff} = 0.22$, $SE = 0.08$, $t = 2.65$, $p_c = .024$, 95% CI [0.02, 0.42]. Neutral faces also tended to have a higher P1 amplitude than fearful faces, $M_{diff} = 0.18$, $SE = 0.08$, $t = 2.13$, $p_c = .066$, 95% CI [–0.02, 0.38]. There was no difference in P1 amplitude between happy and neutral faces, $M_{diff} = 0.04$, $SE = 0.08$, $t = 0.52$, $p_c = .603$, 95% CI [–0.16, 0.25].

Table S2.

Linear Mixed Model for N170 (145 – 175 ms).

Effect	df, df_{error}	*F*	*p*
Status	1, 32884.13	0.50	.478
Expression	2, 138.08	39.49	<.001
Gender (Face)	1, 39.39	2.09	.156
Gender	1, 98.16	0.01	.934
Status × Expression	2, 32883.44	0.40	.670
Status × Gender (Face)	1, 32880.77	0.50	.481
Expression × Gender (Face)	2, 32880.49	0.89	.409
Status × Gender	1, 32883.45	0.43	.510
Expression × Gender	2, 138.07	2.16	.119
Gender (Face) × Gender	1, 124.29	0.00	.988
Status × Expression × Gender (Face)	2, 32880.64	0.17	.847
Status × Expression × Gender	2, 32882.65	0.29	.748
Status × Gender (Face) × Gender	1, 32880.08	0.27	.606
Expression × Gender (Face) × Gender	2, 32877.57	1.28	.277
Status × Expression × Gender (Face) × Gender	2, 32879.84	3.27	.038

For N170, there was a significant main effect of *Expression*, $F(2,138) = 39.49$, $p < .001$. Happy faces elicited a larger (more negative) N170 amplitude compared to neutral faces, M_{diff} = –0.56, *SE* = 0.09, *t* = –6.22, p_c < .001, 95% CI [–0.78, –0.34]. Fearful faces also had a larger N170 amplitude than neutral faces, M_{diff} = –0.73, *SE* = 0.09, *t* = –8.49, p_c < .001, 95% CI [–0.93, –0.52]. Fearful faces also tended to have a larger N170 amplitude than happy faces, M_{diff} = –0.17, *SE* = 0.09, *t* = –1.81, p_c < .073, 95% CI [–0.39, 0.06].

Table S3.

Linear Mixed Model for LPP (300 – 600 ms).

Effect	df, df_{error}	*F*	*p*
Status	1, 98.22	176.91	<.001
Expression	2, 119.73	68.84	<.001
Gender (Face)	1, 41.81	8.33	.006
Gender	1, 98.00	12.48	<.001
Status × Expression	2, 32870.52	3.97	.019
Status × Gender (Face)	1, 32861.92	0.77	.381
Expression × Gender (Face)	2, 32864.39	1.11	.331
Status × Gender	1, 98.22	12.06	<.001
Expression × Gender	2, 119.72	0.84	.434
Gender (Face) × Gender	1, 32860.14	0.14	.712
Status × Expression × Gender (Face)	2, 32861.73	6.44	.002
Status × Expression × Gender	2, 32869.60	1.06	.347
Status × Gender (Face) × Gender	1, 32861.11	2.26	.133
Expression × Gender (Face) × Gender	2, 32861.04	0.90	.408
Status × Expression × Gender (Face) × Gender	2, 32860.80	2.37	.093

For LPP, there was a main effect of *Expression*, $F(2,120) = 68.84$, $p < .001$. Fearful faces elicited a larger LPP amplitude compared to happy faces, $M_{diff} = 0.97$, $SE = 0.08$, $t = 11.58$, $p_c < .001$, 95% CI [0.77, 1.17]. Neutral faces had a higher LPP amplitude than happy faces, $M_{diff} = 0.60$, $SE = 0.08$, $t = 7.56$, $p_c < .001$, 95% CI [0.40, 0.79]. Fearful faces had a larger LPP amplitude than neutral faces, $M_{diff} = 0.37$, $SE = 0.08$, $t = 4.65$, $p_c < .001$, 95% CI [0.18, 0.57].

There was a significant main effect of *Status*, $F(1,98) = 176.91$, $p < .001$. Target faces elicited a larger LPP amplitude compared to nontarget faces, $M_{diff} = 1.84$, $SE = 0.14$, $t = 13.30$, $p < .001$, 95% CI [1.56, 2.11].

There was a significant interaction between *Expression* and *Status*, $F(2,32871) = 3.97$, $p = .019$. Among target faces, fearful faces elicited a larger LPP amplitude compared to happy faces, $M_{diff} = 0.77$, $SE = 0.13$, $t = 6.09$, $p_c < .001$, 95% CI [0.47, 1.07] but not compared to neutral faces, $M_{diff} = 0.23$, $SE = 0.12$, $t = 1.89$, $p_c = .059$, 95% CI [–0.06, 0.53]. Neutral faces also had a larger LPP amplitude compared to happy faces, $M_{diff} = 0.53$, $SE = 0.12$, $t = 4.33$, $p_c < .001$, 95% CI [0.24, 0.82]. Among nontarget faces, fearful faces elicited a larger LPP amplitude compared to happy faces, $M_{diff} = 1.17$, $SE = 0.09$, $t = 12.49$, $p_c < .001$, 95% CI [0.94, 1.40] and compared to neutral faces, $M_{diff} = 0.51$, $SE = 0.09$, $t = 5.65$, $p_c < .001$, 95% CI [0.29, 0.73]. Neutral faces also had a larger LPP amplitude compared to happy faces, $M_{diff} = 0.66$, $SE = 0.09$, $t = 7.39$, $p_c < .001$, 95% CI [0.44, 0.87].
